# Venous Thromboembolism in Critically Ill Cirrhotic Patients: Practices of Prophylaxis and Incidence

**DOI:** 10.1155/2013/807526

**Published:** 2013-12-10

**Authors:** Hasan M. Al-Dorzi, Hani M. Tamim, Abdulaziz S. Aldawood, Yaseen M. Arabi

**Affiliations:** ^1^Intensive Care Department, King Abdulaziz Medical City, Mail Code 1425, P.O. Box 22490, Riyadh 11426, Saudi Arabia; ^2^College of Medicine, King Saud Bin Abdulaziz University for Health Sciences, Riyadh 11426, Saudi Arabia; ^3^Epidemiology and Biostatistics, King Abdullah International Medical Research Center, King Saud Bin Abdulaziz University for Health Sciences, P.O. Box 22490, Riyadh 11426, Saudi Arabia; ^4^Department of Internal Medicine, American University of Beirut Medical Center, P.O. Box 11-0236, Beirut 1107 2020, Lebanon

## Abstract

*Objectives*. We compared venous thromboembolism (VTE) prophylaxis practices and incidence in critically ill cirrhotic versus noncirrhotic patients and evaluated cirrhosis as a VTE risk factor. *Methods*. A cohort of 798 critically ill patients followed for the development of clinically detected VTE were categorized according to the diagnosis of cirrhosis. VTE prophylaxis practices and incidence were compared. *Results*. Seventy-five (9.4%) patients had cirrhosis with significantly higher INR (2.2 ± 0.9 versus 1.3 ± 0.6, *P* < 0.0001), lower platelet counts (115,000 ± 90,000 versus 258,000 ± 155,000/**μ**L, *P* < 0.0001), and higher creatinine compared to noncirrhotic patients. Among cirrhotics, 31 patients received only mechanical prophylaxis, 24 received pharmacologic prophylaxis, and 20 did not have any prophylaxis. Cirrhotic patients were less likely to receive pharmacologic prophylaxis (odds ratio, 0.08; 95% confidence interval (CI), 0.04–0.14). VTE occurred in only two (2.7%) cirrhotic patients compared to 7.6% in noncirrhotic patients (*P* = 0.11). The incidence rate was 2.2 events per 1000 patient-ICU days for cirrhotic patients and 3.6 events per 1000 patient-ICU days for noncirrhotics (incidence rate ratio, 0.61; 95% CI, 0.15–2.52). On multivariate Cox regression analysis, cirrhosis was not associated with VTE risk (hazard ratio, 0.40; 95% CI, 0.10–1.67). *Conclusions*. In critically ill cirrhotic patients, VTE incidence did not statistically differ from that in noncirrhotic patients.

## 1. Background

Chronic liver disease leads to decreased synthesis of coagulation proteins, such as factors II, VII, IX, and X, and is frequently associated with thrombocytopenia [1–3]. Whether these abnormalities make cirrhotic patients less prone to venous thromboembolism (VTE) than the general population is unclear, especially given that cirrhosis is also associated with decreased production of anticoagulation factors, such as protein C, protein S, and antithrombin III [2, 3]. A population-based, case-control study found that liver disease was associated with reduced VTE risk (odds ratio (OR), 0.1; 95% CI, 0.0–0.7) [4]. One retrospective case-control study in hospitalized cirrhotic patients found that VTE occurred in only 0.5% of patients [5], a rate that was lower than that reported in general medical patients [5]. However, more recent studies found higher VTE rates in hospitalized cirrhotic patients (2.7–6.3%) [6, 7]. Additionally, a study of 963 cirrhotic patients and 12,405 controls admitted to a tertiary care hospital found that cirrhotics had higher (1.8%) VTE incidence than controls in general (0.9%, *P* = 0.007) but lower than patients with heart failure (7.8%), chronic kidney disease (7.1%), and cancer (6.1%) [8]. The study also showed that cirrhosis was not associated with VTE on multivariate analysis (OR, 0.87; 95% CI, 0.28–2.63) [8]. None of these studies evaluated VTE exclusively in critically ill cirrhotic patients, who are likely to have higher VTE risk like other intensive care unit (ICU) patients [9]. In addition, the evidence-based VTE prophylaxis guidelines have no separate recommendation for cirrhotic patients [10–12] and caution against pharmacologic prophylaxis for patients with bleeding risk including those with platelet count < 50,000/*μ*L, liver failure, and international normalized ratio (INR) > 1.5 [12]. This may make the practices of VTE prophylaxis in cirrhotic patients inconsistent and variable.

The present study described the practices of VTE prophylaxis in cirrhotic patients admitted to the ICU in relation to coagulation status and evaluated the incidence of VTE (deep venous thrombosis (DVT) or pulmonary embolism (PE)) compared to other critically ill patients and studied the outcome of VTE in this patient population.

## 2. Methods

### 2.1. Setting and Patients

This was a retrospective analysis of a prospective cohort of 798 consecutive patients who were admitted to the ICU of King Abdulaziz Medical City, Riyadh, from January 1, 2006, to June 30, 2008, and were followed for the occurrence of DVT or PE from ICU admission till death in the ICU or up to 5 days after ICU discharge if remained hospitalized. Patients with the following conditions were excluded from the cohort: expected ICU stay <48 hours, brain death on admission, Do-Not-Resuscitate order in the first 48 hours of admission, chronic systemic anticoagulation, and DVT/PE on or within 24 hours of ICU admission. The hospital was a 900-bed tertiary care center, was accredited by the Joint Commission International, and had developed its own VTE prophylaxis guidelines, which mostly followed the American College of Chest Physicians evidence-based clinical practice guidelines [10, 13]. The ICU was a 21-bed closed medical surgical unit staffed with board certified intensivists 24 hours a day, 7 days per week [14]. In this ICU, VTE prophylaxis was ordered by the treating intensivist as pharmacologic prophylaxis (unfractionated heparin or low-molecular-weight heparin) and/or mechanical prophylaxis in the form of graduated compression stocking (GCS) and/or intermittent pneumatic compression (IPC) devices as part of admission order set. Investigation for VTE was performed when clinically suspected by the treating intensivist. Venous duplex ultrasound of the extremities was performed to diagnose DVT and helical chest computed tomography to evaluate PE.

### 2.2. Data Collection

In the present study, a trained research physician evaluated and followed the patients in the cohort almost on a daily basis for up to 30 days in the ICU and five days after ICU discharge. The required data, such as laboratory results, VTE prophylaxis practices, and outcomes, were recorded in case report forms. Charts were also reviewed to obtain additional clinical information, such as past medical history and VTE risk factors. Patients known to have liver cirrhosis, based on their documented past medical history, were identified and compared to all other patients for the incidence of VTE (DVT or PE). In addition, the following information was noted: age, gender, body mass index, reason of ICU admission (postoperative or medical), Acute Physiology and Chronic Health Evaluation (APACHE) II score [15], requirement for mechanical ventilation and duration of mechanical ventilation, admission creatinine, bilirubin, ammonia, albumin, platelet count, INR of the prothrombin time and partial thromboplastin time (PTT), use of mechanical prophylaxis in the form of graduated GCS or IPC, use of pharmacologic prophylaxis in the form of unfractionated and low-molecular-weight heparin, and transfusion of blood products. Other studied outcomes included the duration of mechanical ventilation, length of stay in the ICU and the hospital, and ICU and hospital mortality. Patients were also categorized according to clinically relevant cutoffs of platelet count (cutoff of 50,000/*μ*L), INR (cutoff of 2.0), and PTT (cutoff of 45 seconds).

### 2.3. Statistical Analysis

Data were analyzed using SAS software (version 9.0; SAS Institute, Cary, NC, USA). Continuous data were presented as means and standard deviation (SD), whereas categorical variables were summarized as numbers and percentages (%). Chi-square or Fisher's exact test was used to evaluate differences in categorical variables between cirrhotic and noncirrhotic patients. Similarly, Student's *t*-test was used to assess differences in continuous variables. We calculated the incidence rates for VTE in cirrhotic and noncirrhotics and reported the incidence rate ratio with 95% confidence interval (CI). Cox proportional regression analysis was performed to determine if cirrhosis was an independent risk factor for VTE in critically ill patients adjusting for clinically significant factors and imbalances in baseline characteristics, which were age, gender, creatinine, use of low-molecular-weight heparin, platelet count, INR, admission diagnosis, trauma, femur fracture, presence of central line, sepsis, spinal cord injury status, malignancy, surgery, previous PE or DVT, and stroke. Its result was presented as hazard ratio with 95% CI.

## 3. Results

### 3.1. General Characteristics and VTE Risk Factors

Out of the 798 patients included in the cohort, 75 (9.4%) had liver cirrhosis.  Table 1 describes their characteristics and VTE risk factors compared to noncirrhotic patients. Patients with cirrhosis were older, had higher APACHE II score (30 ± 8 versus 23 ± 9, *P* < 0.0001), and were less likely to be admitted postoperatively or after polytrauma. In addition, they were less likely to have a diagnosis of stroke and to be functionally independent and mobile at home before admission. However, they had similar prevalence of malignancy and requirement for mechanical ventilation. On admission to the ICU, they also had significantly higher INR (2.2 ± 0.9 versus 1.3 ± 0.6, *P* < 0.0001), lower platelet count (115,000 ± 90,000 versus 258,000 ± 155,000/*μ*L, *P* < 0.0001), and higher serum bilirubin and creatinine.

### 3.2. Practices of VTE Prophylaxis

Table 2 summarizes the practices of VTE prophylaxis according to cirrhosis status. Forty-three (57%) cirrhotic patients received mechanical prophylaxis in the form of IPC and/or GCS, with 31 (41%) cirrhotic patients receiving only mechanical prophylaxis. Use of mechanical prophylaxis was similar in cirrhotic and noncirrhotic patients (57.3% and 50.6%, resp., *P* = 0.27) (OR, 1.31; 95% CI, 0.81–2.12). However, more (41.3%) cirrhotics received only mechanical prophylaxis compared to noncirrhotics (7.7%, *P* < 0.0001). For pharmacologic prophylaxis, cirrhotic patients were less likely to receive pharmacologic prophylaxis for DVT prophylaxis (odds ratio, 0.08; 95% CI, 0.04–0.14) such that only 22 (29.3%) patients received unfractionated heparin compared to 66.3% in noncirrhotic patients (*P* < 0.0001) and two (2.7%) patients received low-molecular-weight heparin compared to 31.1% of noncirrhotic patients (*P* < 0.0001). Of note, more (26.7%) cirrhotic patients did not receive any form of prophylaxis compared to noncirrhotics (7.2%, *P* < 0.0001). Moreover, cirrhotic patients had shorter duration of pharmacologic prophylaxis (5.1 ± 4.2 versus 10.4 ± 7.6 days for noncirrhotics, *P* < 0.0001) and more days in the ICU without pharmacologic prophylaxis (8.4 ± 7.0 versus 4.1 ± 6.1 days for noncirrhotics, *P* < 0.0001).

**Figure 1:**
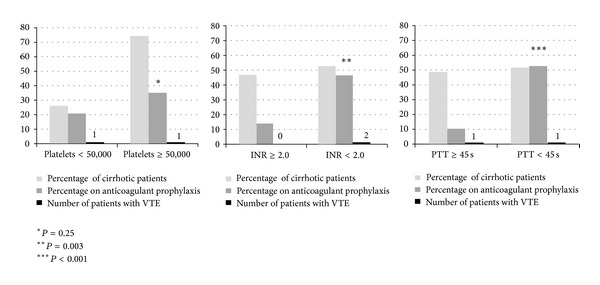
The relationship between the use of pharmacologic prophylaxis and the admission values of platelet count (per *μ*L), INR, and PTT in cirrhotic patients.

Figure 1 describes the use of pharmacologic prophylaxis in cirrhotic patients according to platelet count (< versus ≥50,000/*μ*L), INR (< versus ≥2.0), and PTT (< versus ≥45 seconds) on ICU admission. Pharmacologic prophylaxis was used less often in patients with INR ≥ 2.0 (*P* = 0.003) and PTT ≥ 45 seconds (*P* < 0.001). In cirrhotic patients, those who were given pharmacologic prophylaxis had higher platelet counts (146,000 ± 100,000/*μ*L versus 99,000 ± 82,000/*μ*L, *P* = 0.04) and lower PTT (39 ± 11 seconds versus 61 ± 41 seconds, *P* = 0.0008) and INR (1.7 ± 0.5 versus 2.4 ± 1.1, *P* = 0.003) and received less transfusion of blood products (RBC transfusion: 2.2 ± 2.8 units versus 5.3 ± 5.7 units, *P* = 0.002; platelet transfusion: 5.0 ± 9.0 units versus 12.9 ± 20.9 units, *P* = 0.02; fresh frozen plasma: 7.1 ± 9.2 units versus 15.2 ± 12.3 units, *P* = 0.006; and cryoprecipitates: 1.1 ± 3.0 units versus 4.7 ± 8.2 units, *P* = 0.007) compared to patients not given pharmacologic prophylaxis.

The most common reason for not using pharmacologic prophylaxis in cirrhotic patients was bleeding risk in 55 (73.3%) patients. In noncirrhotic patients, the reasons were more variable and included recent surgery in 137 (18.9%) patients, intracranial hemorrhage in 123 (17.0%) patients, and other bleeding risks in 142 (19.6%) patients. The most common reasons for not using mechanical prophylaxis were the use of pharmacologic prophylaxis in 12 cirrhotic and 327 noncirrhotic patients and lower extremity fracture in 21 noncirrhotic patients.

### 3.3. Incidence of VTE during ICU Stay

Table 3 describes the outcomes of patients according to liver cirrhosis status. VTE was diagnosed in only two (2.7%) cirrhotic patients compared to 55 (7.6%) noncirrhotic patients (*P* = 0.11). On multivariate Cox regression analysis, cirrhosis was not an independent risk factor for VTE (hazard ratio, 0.40; 95% CI, 0.10–1.67). The incidence rate ratio was 2.2 events per 1000 patient-days for cirrhotic patients and 3.6 events per 1000 patient-days for noncirrhotics yielding an incidence rate ratio of 0.61 (95% CI, 0.15–2.52). The two cirrhotic patients who had VTE were females with mean INR = 1.25 (1.1 and 1.4) and both had femoral central lines. One had DVT on the fifth ICU day and the other on the 13th day. For VTE prophylaxis, one was on unfractionated heparin with normal platelet count and the other was only on mechanical prophylaxis because of platelet count < 50,000/*μ*L. None of the cirrhotic patients was diagnosed to have PE compared to 28 (3.9%) patients without cirrhosis (*P* = 0.08).

Of note, patients with liver cirrhosis had significantly shorter duration of stay in the ICU (10 ± 8 versus 17 ± 31 days) and higher mortality in the ICU (64% versus 17%, *P* < 0.0001 and hospital (80% versus 31%, *P* < 0.0001)). The two cirrhotic patients who had DVT died in the ICU.

## 4. Discussion

The main findings of the present study were the following: the majority (73%) of critically ill cirrhotic patients received at least one form of VTE prophylaxis, but they were less likely to receive pharmacologic prophylaxis than noncirrhotic patients; VTE incidence did not differ between cirrhotic and noncirrhotic critically ill patients when VTE was investigated upon clinical suspicion and cirrhosis was not an independent predictor of the occurrence of VTE in the ICU.

Cirrhotic patients have decreased synthesis of procoagulant proteins, which results in the prolongation of the prothrombin time, and frequently have thrombocytopenia [1–3]. Hence, they are frequently considered to be autoanticoagulated. However, these patients have a concomitant reduced synthesis of anticoagulant factors [2, 3], may experience poor blood flow and vasculopathy [2, 3], and have high incidence of antiphospholipid antibodies [16], all of which increase thrombosis risk. Studies on thromboprophylaxis in cirrhotic patients are lacking. In fact, severe liver disease was an exclusion criterion for many thromboprophylaxis trials in medical patients [17–19]. This may have resulted in evidence-based VTE prophylaxis guidelines having no separate recommendations for patients with liver cirrhosis [10–12, 20], although they are frequently considered as patients with increased bleeding risk [11, 12]. This may make clinicians reluctant to use pharmacologic VTE prophylaxis. This was observed in multiple studies. Northup and colleagues found that only 21% of 113 hospitalized cirrhotic patients received DVT prophylaxis [5]. Dabbagh and colleagues similarly found that only 25.3% of 190 hospitalized cirrhotic patients received DVT prophylaxis (9% pharmacologic and 16.3% mechanical) [7]. In a previous study of 226 cirrhotic patients admitted to our hospital, approximately 76% of the cirrhotic patients received neither pharmacologic nor mechanical DVT prophylaxis [6]. The present study evaluated cirrhotic patients who developed critical illness and were managed with interventions that further increase VTE risk. We have found that the practices of VTE prophylaxis in cirrhotic ICU patients were significantly different from those in noncirrhotics. In contrast to other studies, 73% of cirrhotic patients received at least one form of prophylaxis in our study. Pharmacologic prophylaxis was used less often especially in patients with higher INR and PTT. This was also observed in other studies.

Studies that evaluated VTE incidence in cirrhotic patients showed variable findings. A population-based case-control study over 15 years between 1976 and 1990 found that serious liver disease was associated with a 90% decrease in the risk for VTE [4]. Northup et al. conducted a retrospective cohort study over an eight-year period, identified 113 hospitalized patients with cirrhosis, and found a VTE incidence of 0.5% [5]. Gulley et al. conducted a case-control study of medical patients at a tertiary care hospital, identified patients with newly diagnosed VTE based on the related ICD-9 codes, and confirmed the diagnosis by positive computerized tomography, positive Doppler ultrasound of the extremities, and/or high probability V/Q scan [8]. They found a 1.8% incidence of VTE in cirrhotic patients compared to 0.9% in the controls [8]. However, other studies have found higher VTE incidence in hospitalized cirrhotic patients [7, 21]. In a recently published observational study of 190 hospitalized cirrhotic patients admitted to a tertiary university hospital over a seven-year period, the incidence of new VTE was 6.3% [7]. In a study from Asia where viral hepatitis is the predominant cause of cirrhosis, the incidence of VTE in hospitalized cirrhotic patients was 4.7% [21]. In a study from our hospital, 2.7% of 226 cirrhotic patients admitted over one-year period developed VTE, all of which were DVT [6]. The present study was on a different population and for a different follow-up period and found an incidence rate of 2.2 VTE events per 1000 patient-days for cirrhotic patients. This incidence rate was not significantly different than that of noncirrhotic patients. We found that cirrhotic patients who got pharmacologic prophylaxis had higher platelet count and more favorable coagulation profile than cirrhotic patients without pharmacologic prophylaxis suggesting that these factors are important when clinicians decide on the VTE prophylaxis method. Additionally, the same patients had lower needs for blood transfusion during ICU stay, possibly for the same reasons.

The majority of critically ill patients have multiple VTE risk factors [22]. Most of these risk factors apply to cirrhotic patients during critical illness. PTT and serum albumin have been found to independently predict VTE in cirrhotic patients [8]. However, Dabbagh and colleagues showed that the level of INR did not affect VTE incidence [7]. In the present study, the only two cirrhotic patients who developed VTE had their INR within the normal range (<1.5). They also had femoral central venous catheters, which increases thrombosis risk through multiple mechanisms [23]. It is not surprising that the femoral vein was frequently used for central venous access in cirrhotic patients as these patients were thought to be at high risk of bleeding. The femoral route has several potential advantages. The site is compressible if bleeding occurs and it can facilitate successful resuscitation in acute decompensation [24].

The present study has strengths and limitations. Among the strengths are the prospective data collection by a trained research physician and the presence of evidence-based hospital VTE prophylaxis guidelines and order set. Limitations include the post hoc analysis, the small number of patients with cirrhosis, the low number of VTE events in this group, conducting the study at a single center and investigating VTE only when it was clinically suspected, which may have led to diagnosis bias. It is possible that physicians were less likely to suspect VTE or perform workup in cirrhotic patients because of the perception that these patients have low likelihood of having VTE due to coagulopathy and also due to poor prognosis. Such bias leads to underestimation of the true VTE risk in cirrhotic patients. So subclinical VTE might have been missed. The incidence of VTE in our cohort of 798 consecutive critically ill patients was 7.1%. Other prospective ICU cohorts have shown different VTE incidence rates [25–27]. Hirsch et al. performed Doppler ultrasound imaging twice weekly in the ICU and once within one week of ICU discharge, had only 61% of patients given prophylaxis, and found a DVT incidence of 33% [26]. In Ibrahim et al.'s study, Doppler ultrasound of the lower and upper extremities was performed every seven days, all patients received VTE prophylaxis, and the DVT incidence was 23.6% [27]. Cook et al. performed Doppler ultrasound of lower extremities within 48 hours of ICU admission and then twice weekly thereafter, used VTE prophylaxis for all patients (pharmacologic prophylaxis for 92.8% and mechanical for the other 7.2%), and found a DVT incidence of 9.6% [25]. The difference in DVT incidence between the present study and the other cohort studies is mainly related to the fact that VTE evaluation was performed when clinically suspected in the present study compared to scheduled DVT surveillance in the other studies.

In conclusion, we have found that pharmacologic prophylaxis was administered less often in critically ill cirrhotic patients compared to noncirrhotic patients, but 73% of cirrhotic patients received at least one form of VTE prophylaxis. We also found that VTE incidence in cirrhotic patients was not statistically different from that of other critically ill patients. We think that all critically ill cirrhotic patients should receive VTE prophylaxis. Those with important risk factors for bleeding should receive mechanical prophylaxis, while others can be considered for pharmacologic prophylaxis after careful consideration of bleeding risk.

## Figures and Tables

**Table 1:** Characteristics of patients with and without liver cirrhosis.

	Cirrhotic patients *N* = 75	Noncirrhotic patients *N* = 723	*P* value
Age (years), mean ± SD	58.8 ± 14.3	49.3 ± 21.6	<0.0001
Male gender, *N* (%)	37 (49.3)	498 (68.9)	0.0006
Body mass index (Kg/m^2^), mean ± SD	29.4 ± 7.8	28.4 ± 10.4	0.31
APACHE II score, mean ± SD	29.6 ± 8.2	23.4 ± 8.9	<0.0001
Admission GCS score, mean ± SD	9.2 ± 4.1	8.5 ± 4.1	0.19
Reason for ICU admission:			<0.0001
Respiratory, *N* (%)	10 (13.3)	159 (22.0)	
Cardiovascular, *N* (%)	45 (60.0)	201 (27.8)	
Neurological, *N* (%)	1 (1.3)	56 (7.8)	
Other medical, *N* (%)	17 (22.7)	19 (2.6)	
Nonoperative trauma, *N* (%)	1 (1.3)	122 (16.9)	
Postoperative, *N* (%)	1 (1.3)	166 (23.0)	
Bedridden before admission, *N* (%)	54 (72.0)	340 (47.0)	<0.0001
Congestive heart failure, *N* (%)	2 (2.6)	36 (5.0)	0.57
Previous stroke, *N* (%)	4 (5.3)	102 (14.1)	0.03
Active malignancy, *N* (%)	7 (9.3)	87 (12.0)	0.49
Femur fracture, *N* (%)	1 (1.3)	51 (7.0)	0.08
Spinal cord injury, *N* (%)	0 (0)	20 (2.8)	0.24
Previous history of VTE, *N* (%)	0 (0)	12 (1.7)	0.62
Sepsis on admission, *N* (%)	55 (73.3)	266 (36.8)	<0.0001
Mechanical ventilation on admission, *N* (%)	62 (82.7)	625 (86.4)	0.37
Femoral central venous catheter, *N* (%)	47 (62.7)	295 (40.8)	0.0003
Internal jugular or subclavian central venous catheter, *N* (%)	50 (66.7)	475 (65.7)	0.87
Admission creatinine (*μ*mol/L), mean ± SD	208 ± 149	154 ± 143	0.002
Admission lactate (mmol/L), mean ± SD	4.5 ± 3.8	3.1 ± 3.1	0.004
Admission bilirubin (*μ*mol/L), mean ± SD	261 ± 272	34 ± 62	<0.0001
Admission platelet count/*μ*L, mean ± SD	115,000 ± 90,000	258,000 ± 155,000	<0.0001
Admission INR, mean ± SD	2.2 ± 0.9	1.3 ± 0.6	<0.0001
Admission PTT (seconds), mean ± SD	44.1 ± 62.0	54.3 ± 35.9	0.07

APACHE: Acute Physiology and Chronic Health Evaluation; GCS: Glasgow Coma Scale; ICU: intensive care unit; INR: International Normalized Ratio; PTT: partial thromboplastin time; SD: standard deviation.

**Table 2:** Practices of VTE prophylaxis in cirrhotics and noncirrhotic critically ill patients.

	Cirrhotic patients *N* = 75	Noncirrhotic patients *N* = 723	*P* value
Use of mechanical prophylaxis, *N* (%)	43 (57.3)	366 (50.6)	0.27
Intermittent pneumatic compression	23 (30.6)	232 (32.1)	0.86
Graduated compression stockings	22 (29.3)	175 (24.2)	0.29
Mechanical prophylaxis only	31 (41.3)	56 (7.7)	<0.0001
Use of pharmacologic prophylaxis, *N* (%)	24 (32.0)	615 (85.1)	<0.0001
Unfractionated heparin	22 (29.3)	479 (66.3)	<0.0001
Low-molecular-weight heparin	2 (2.7)	225 (31.1)	<0.0001
Pharmacologic prophylaxis only	12 (16.0)	305 (42.2)	<0.0001
No VTE prophylaxis, *N* (%)	20 (26.7)	52 (7.2)	<0.0001
Use of both mechanical and pharmacologic prophylaxis simultaneously, *N* (%)	12 (16.0)	310 (42.9)	<0.0001
Duration of pharmacologic prophylaxis (days)^∗¶^, mean ± SD	5.1 ± 4.2	10.4 ± 7.6	<0.0001
Duration of mechanical prophylaxis (days), mean ± SD			
Intermittent pneumatic compression	2.4 ± 4.7	3.3 ± 6.3	0.13
Graduated compression stockings	2.9 ± 5.4	2.3 ± 5.2	0.41
Stay in the ICU without pharmacologic prophylaxis^¶^ (days), mean ± SD	8.4 ± 7.0	4.1 ± 6.1	<0.0001

ICU: intensive care unit; SD: standard deviation.

*For patients who received pharmacologic prophylaxis.

^¶^Data on thromboprophylaxis were obtained for a maximum of 30 days of ICU stay.

**Table 3:** Outcomes of patients in the cohort according to cirrhosis status.

	Cirrhotic patients *N* = 75	Noncirrhotic patients *N* = 723	*P* value
Venous thromboembolism, *N* (%)	2 (2.7)	55 (7.6)	0.11
Deep venous thrombosis alone	2 (2.7)	27 (3.7)	0.64
Pulmonary embolism alone	0 (0)	24 (3.3)	0.11
Deep venous thrombosis and pulmonary embolism	0 (0)	4 (0.6)	0.52
Duration of MV (days)	8.3 ± 6.9	9.9 ± 12.7	0.09
ICU LOS (days)	10.2 ± 7.7	16.9 ± 31.3	<0.0001
Hospital LOS (days)	27.7 ± 21.2	75.9 ± 125.1	<0.0001
ICU mortality, *N* (%)	48 (64.0)	123 (17.0)	<0.0001
Hospital mortality, *N* (%)	60 (80.0)	226 (31.3)	<0.0001

ICU: intensive care unit; LOS: length of stay; MV: mechanical ventilation.
